# Current Understanding of Neurofibromatosis Type 1, 2, and Schwannomatosis

**DOI:** 10.3390/ijms22115850

**Published:** 2021-05-29

**Authors:** Ryota Tamura

**Affiliations:** Department of Neurosurgery, Kawasaki Municipal Hospital, Shinkawadori, Kanagawa, Kawasaki-ku 210-0013, Japan; moltobello-r-610@keio.jp

**Keywords:** neurofibromatosis type 1, neurofibromatosis type 2, schwannomatosis, molecular targeted therapy, clinical trial

## Abstract

Neurofibromatosis (NF) is a neurocutaneous syndrome characterized by the development of tumors of the central or peripheral nervous system including the brain, spinal cord, organs, skin, and bones. There are three types of NF: NF1 accounting for 96% of all cases, NF2 in 3%, and schwannomatosis (SWN) in <1%. The NF1 gene is located on chromosome 17q11.2, which encodes for a tumor suppressor protein, neurofibromin, that functions as a negative regulator of Ras/MAPK and PI3K/mTOR signaling pathways. The NF2 gene is identified on chromosome 22q12, which encodes for merlin, a tumor suppressor protein related to ezrin-radixin-moesin that modulates the activity of PI3K/AKT, Raf/MEK/ERK, and mTOR signaling pathways. In contrast, molecular insights on the different forms of SWN remain unclear. Inactivating mutations in the tumor suppressor genes SMARCB1 and LZTR1 are considered responsible for a majority of cases. Recently, treatment strategies to target specific genetic or molecular events involved in their tumorigenesis are developed. This study discusses molecular pathways and related targeted therapies for NF1, NF2, and SWN and reviews recent clinical trials which involve NF patients.

## 1. Introduction

Neurofibromatosis (NF) is a genetic disorder that causes multiple tumors on nerve tissues, including brain, spinal cord, and peripheral nerves [1,2,3]. There are three types in NF: NF1, NF2, and schwannomatosis (SWN) [4]. NF1 is the most prevalent, accounting for 96% of all cases and characterized by neurofibromas (peripheral nerve tumors) that induce skin changes and bone deformation. Characterized by tumors originating from Schwann cells, NF2 and SWN are rare compared to NF1, occurring in 3% and <1%, respectively. NF2 typically causes hearing loss and vestibular dysfunction [5,6]. Whereas, SWN causes intense pain [5,6].

Different mutations result in the three types of NF. Currently, there is no way to prevent or cure these diseases. In this review, we discuss the clinical, genetic, and molecular characteristics of NF and the current molecular targeted therapies, and review the recent clinical trials for the patients with NF.

## 2. Neurofibromatosis Type 1

### 2.1. Clinical Characteristics

NF1, which is known as von Recklinghausen’s disease, causes various manifestations such as multiple flat, light-brown patches of skin pigment (café-au-lait spots), skinfold freckling, visible neurofibromas under the skin, and small nodules of the iris (Lisch nodules) (Table 1) [7,8,9,10]. NF1 occurs in 1 in 3000–4000 people worldwide. Although NF1 is inherited via autosomal dominance, 50% of detected mutations are de novo [7,8,9,10,11]. The condition typically progresses over time since childhood. It has been shown that NF1 patients have decreased life expectancy of 15 years compared to the general population [12]. Malignant tumors and vascular disease have been significantly associated with the death of NF1 patients aged <40 years [13].

### 2.2. Genetic and Molecular Characteristics

**Figure 1:**
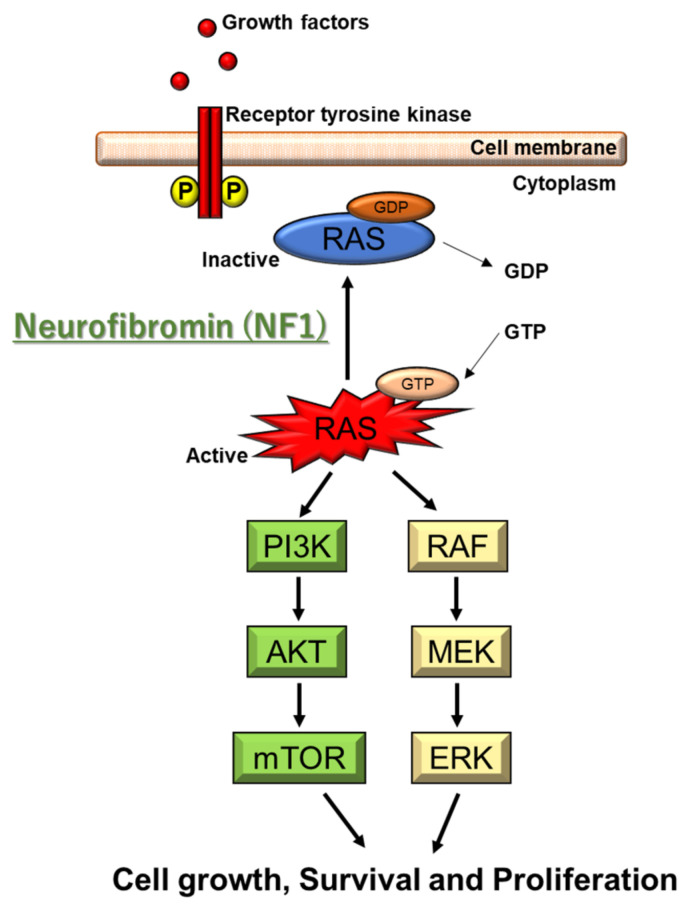
Molecular pathogenesis of NF1. NF1 codes for neurofibromin, which is a Ras-GTPase-activating protein (Ras-GAP). NF1 gene deficiency leads to Ras hyperactivation, which causes the subsequent activation of the AKT/mTOR and Raf/MEK/ERK pathways. ERK activates SYN1 modulating GABA release. Ras-GTP also activates Rac1 and Cdc42 pathways, leading to overactivation of PAK1.

The NF1 gene is located on 17q11.2 of chromosome 17. The point mutations are responsible for 90% of NF1 patients. A single exon or whole NF1 gene deletion is associated with the remaining 5–7% [14,15]. NF1 codes for neurofibromin, which is a Ras-GTPase-activating protein (Ras-GAP) [15]. Neurofibromin protein is produced in nerve cells, oligodendrocytes, and Schwann cells. NF1 gene deficiency leads to Ras hyperactivation, leading to the subsequent activation of the AKT/mTOR and Raf/MEK/ERK pathways [15] (Figure 1). ERK activates SYN1, modulating GABA release. Ras-GTP also activates Rac1 and Cdc42 pathways, leading to overactivation of PAK1 [16,17]. Nonfunctional neurofibromin protein influences the growth of neurofibromas along the nerves of the whole body. However, it currently remains unclear how NF1 gene mutations cause café-au-lait spots and learning disabilities.

Several thousand pathogenic NF1 variants have been identified in NF1 patients [18]. Some patients who have a higher incidence of intellectual disability, developmental delay, dysmorphic facial features, and earlier appearance of cutaneous neurofibromas tend to have malignant peripheral nerve sheath tumors (MPNSTs) [19,20,21]. The c.2970–2972 delAAT (p.M992del) mutation is associated with a mild phenotype [22]. A severe phenotype including plexiform neurofibromas, spinal neurofibromas, optic glioma, skeletal dysplasia, and malignant transformation is associated with missense variants in codons 844 to 848 [23]; p.Met1149, p.Arg1276, or p.Lys1423 missense variants with a Noonan syndrome-like phenotype [24]; and p.Arg1276 and p.Met1149 with spinal neurofibromas and café-au-lait macules, respectively [14].

Furthermore, NF1 can be associated with specific genetic lesions. NF1 gene product acts as a negative regulator of the product of RAS genes which are activated in myelodysplastic syndromes and acute myeloid leukemia through point mutations [25].

Genetic testing can be performed to confirm the diagnosis and to assist the direct screening of family members [26]. However, a negative test does not completely exclude the diagnosis as it may also represent mosaicism for a pathogenic variant. NF1 is noted for the considerable inter and intrafamilial variation observed in the clinical phenotype, even in patients who share the same germline mutation. This variability poses disease prediction and management problems. Allelic heterogeneity may be a possible cause of the multiple phenotypes in NF1 [27]. Meanwhile, a positive NF1 mutation does not predict the severity of the disease. In general, targeted testing is performed to detect the mutation rather than comprehensively analyze the mutation of the entire gene. The development of next-generation sequencing technologies allows for rapid diagnosis of NF1 [28]. Amniocentesis or chorionic villus sampling can be performed to obtain a sample for genotyping the fetus if the precise mutation of an affected NF1 family member is detected [28].

### 2.3. Therapeutic Strategies

Surgery is the principal mode of treatment for neurofibromas, but comes with a high recurrence rate after partial removal of large plexiform neurofibromas. In the case of NF1-related tumors, there is no consensus with regard to the treatment strategy due to the multiple pathways involved in the growth of NF1-related tumors. Targeted therapy can show a great impact [17,29]. Anti-Ras therapies are ideal because Ras-GTP is upregulated in neurofibromas. Agents targeting Ras signaling and other pathways (tipifarnib, pirfenidone, sirolimus, pegylated interferon alfa-2b, and imatinib) have been used for plexiform neurofibromas in phase II clinical trials. Targeting downstream effectors of the Ras signaling pathway, such as agents inhibiting MEK and PI3K, and the pharmacological inhibition of kit activity and α4β1 adhesion are considered promising therapeutic strategies. A new clinical trial suggests that the MEK inhibitor selumetinib induces partial responses in children with NF1 who have inoperable plexiform neurofibromas [30]. In April 2020, the US Food and Drug Administration approved selumetinib (KOSELUGO, AstraZeneca) for pediatric NF1 patients aged at least 2 years who have symptomatic, inoperable plexiform neurofibromas [31]. Rapamycin is an inhibitor of the mTOR pathway, but induces AKT activation, thus demonstrating both therapeutic potential and limits [17,29].

Low grade optic gliomas are the most common central nervous system tumors in NF1 patients [32], with pilocytic astrocytoma known as its indolent subtype. Regimens involving carboplatin and vincristine are the most frequently used chemotherapy for optic nerve glioma [33].

### 2.4. Ongoing Clinical Trials

Table 2 shows ongoing phase I/II clinical trials for NF1 patients using various molecular targeted agents such as selumetinib and mirdametinib (MEK inhibitor), binimetinib and trametinib (MEK1/2 inhibitor), and cabozantinib (VEGFR2 inhibitor). The primary outcome is volumetric response, toxicity, and event-free survival.

### 2.5. Animal Models

The development of genetically engineered mouse models of NF1-related tumors has promoted preclinical trials of targeted agents [34,35,36,37]. Initial studies focused on a targeted mutation of the Nf1 gene. Traditional NF1+/- mice were generated in which one allele of the murine Nf1 gene is inactivated by the insertion of a neomycin cassette [38,39,40]. Second-generation models included NF1-/- chimeric mice and NF1 exon-specific knockout mice [41]. Tissue-specific NF1 inactivation can be accomplished by the Cre/LoxP technology, in which LoxP recombinatorial sequences are inserted into noncoding regions of the NF1 gene [40], thus providing important insights into the function of neurofibromin in specific cell types.

Several NF1-associated high-grade glioma models have been established by coupling complete NF1 gene inactivation with loss of other tumor suppressor genes (p53, PTEN), for example, strategies targeting genes such as CRISPR/Cas9 [42], standard and conditional knockout mice [43,44], and viral gene silencing [45]. NF1 optic glioma requires a combination of a germline inactivating NF1 gene mutation and somatic NF1 loss in neuroglial progenitor cells [46].

Recently, NF1 porcine models have been established [47,48]. The anatomical, biochemical, and cellular components of porcine nerves are comparable to humans. This innovative model may recapitulate the wide spectrum of the phenotypic and pathological changes associated with NF1, accelerating NF1 research and therapies.

## 3. Neurofibromatosis Type 2

### 3.1. Clinical Characteristics

NF2 is an autosomal dominantly inherited syndrome that predisposes individuals to multiple nervous tumors. A de novo mutation may take place after fertilization, resulting in a mosaic expression [49,50]. Diagnosis is based on clinical and neuroimaging studies (Table 3). Two large population-based studies reported that this condition occurs in 1 in 25,000 people [51]. The actuarial survival after diagnosis is 15 years, with an average age at death of 36 years [52] and a 10-year survival rate of 67% [53]. NF2 patients uniformly develop schwannomas on the bilateral vestibular portion of the eighth cranial nerve and on other cranial nerves, spinal roots, or peripheral nerves [54]. In addition, NF2 patients often develop multiple meningiomas and ependymomas at an early age.

NF2 patients often experience hearing loss, balance problems, flesh colored skin flaps, and muscle wasting. Some develop mononeuropathy, often involving the facial nerve. Severe polyneuropathy is noted in 3–5% of adult NF2 patients [55]. Visual impairment is likely due to cataracts, optic nerve meningiomas, and retinal hamartomas [56,57,58]. Approximately 70% of NF2 patients have cutaneous manifestations: only 10% have more than 10 skin tumors [54]. Plaque-like lesions may be more pigmented than the surrounding skin with increased hair. Subcutaneous nodules are identified along the peripheral nerve. Intracutaneous schwannomas, which are similar to those observed in NF1 patients, are occasionally seen.

### 3.2. Genetic and Molecular Characteristics

**Figure 2:**
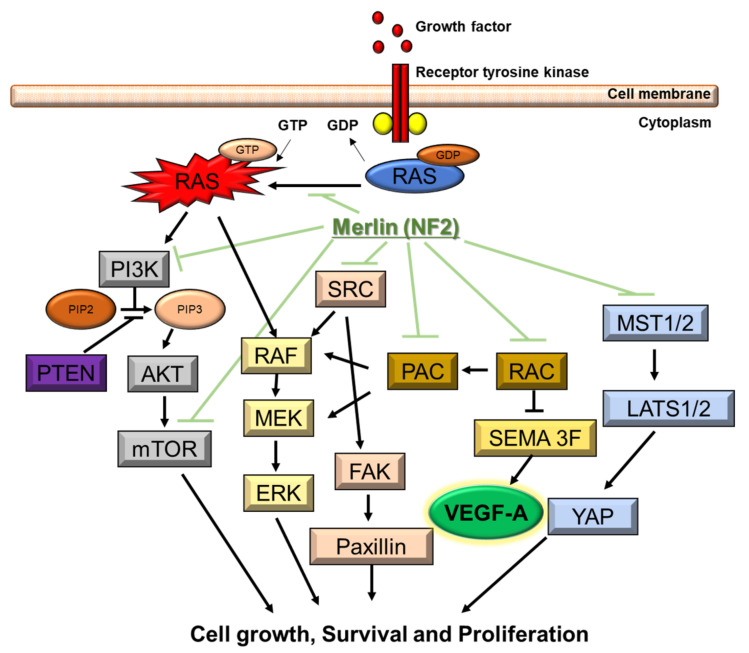
Molecular pathogenesis of NF2. NF2 gene encodes merlin. Merlin regulates multiple proliferative signaling pathways. At the membrane, merlin blocks signaling caused by integrins and tyrosine receptor kinases. Merlin can also inhibit downstream signalings, including the p21-activated kinase signaling, Ras/Raf/MEK/ERK, FAK/Src, PI3K/AKT, Rac/PAK/JNK, mTORC1, and Wnt/β-catenin pathways. Downstream signaling of NF2 includes VEGF-A.

NF2 is caused by a defect in the gene that normally produces merlin, located at 22q12.2 of chromosome 22, which regulates multiple proliferative signaling pathways. At the membrane, merlin blocks signaling caused by integrins and tyrosine receptor kinases. Merlin can also inhibit downstream signalings, including the p21-activated kinase signaling, Ras/Raf/MEK/ERK, FAK/Src, PI3K/AKT, Rac/PAK/JNK, mTORC1, and Wnt/β-catenin pathways (Figure 2). In the nucleus, merlin suppresses the E3 ubiquitin ligase CRL4DCAF1, which also regulates the expression of integrins and tyrosine receptor kinases. The Hippo signaling pathway regulates tissue homeostasis. Merlin is implicated as one of the upstream regulators of the Hippo signaling pathway [49,50,59,60,61].

A de novo mutation results in a mosaic expression. Somatic mosaicism may prevent the molecular diagnosis unless tumor tissue is analyzed [62]. The growth of schwannomas requires inactivation of both NF2 alleles. The “second hit” occurs through loss of the entire NF2 gene and most of chromosome 22. Various types of mutations, such as protein-truncating alterations (frameshift deletions/insertions and nonsense mutations), splice-site mutations, missense mutations, are identified. Truncating mutations (nonsense and frameshifts) are the most frequent germline event and cause the most severe disease. The presence of a truncated protein is associated with younger age at diagnosis and a higher prevalence of meningiomas, spinal tumors, and cranial nerve tumors other than VIII [62]. Deletions in the NH2-terminal domain of merlin proteins are associated with early tumor onset and disease progression [62]. The most common alterations are splice-site mutations or nonsense mutations in exons 1–8. Missense or in-frame deletions have been associated with milder clinical courses. A positional effect with mutations in the latter parts of the gene (exons 14 and 15) is associated with milder disease and fewer meningiomas [63]. Alterations in the conserved N-terminal FERM domain and truncating mutations are typically associated with the Wishart phenotype including younger age at diagnosis, a higher incidence of meningiomas, ophthalmologic and cutaneous lesions, and poor outcomes. Missense and splice-site mutations, particularly in the 3′ end of the gene, are associated with the Gardner phenotype and a better prognosis with fewer meningiomas [64].

The role of mutation screening for NF2 in all patients with a unilateral vestibular schwannoma is less certain. Although these patients show an increased risk for the development of NF2, routine screening for germline mutations is not recommended except in patients younger than 30 years [65,66], but can undergo prenatal diagnosis and pre-implantation genetic diagnosis.

### 3.3. Therapeutic Strategies

To date, there is no established effective treatment for NF2 patients because tumors are highly likely to regrow after surgical resection [67]. Treatment is generally indicated when the patient has risk of brainstem compression, deterioration of hearing, and/or facial nerve dysfunction. Vestibular schwannomas may involve facial nerve fibers, possibly posing a significant risk of damage to the facial nerve during surgery [68]. While the use of stereotactic radiosurgery has recently become an effective management modality for NF2 schwannomas, it is not advocated for multiple or large tumors [69]. Vestibular dysfunction and trigeminal neuropathy have been reported after radiosurgery [70]. Malignant transformation associated with radiosurgery is evidently uncommon [71,72]. Furthermore, surgical resection may be more difficult following stereotactic radiosurgery [69,73].

Vascular endothelial growth factor (VEGF)-A is an important factor for the growth of schwannoma that mainly depends on VEGF-A/VEGF receptor (VEGFR) pathway (not other factors such as estrogen and progesterone) [74,75]. Tumor shrinkage and hearing improvement are identified after administration of bevacizumab (a monoclonal antibody against VEGF-A) in >50% of progressive vestibular schwannomas in NF2 patients. Stable hearing is retained in the majority of the patients [76,77,78,79,80,81,82,83,84]. Bevacizumab is recently considered as a first-line medical therapy for rapidly growing vestibular schwannomas [85]. In a recent meta-analysis of eight observational studies involving 161 patients with NF2-associated vestibular schwannomas, the best response to bevacizumab was partial regression in 41%, no change in 47%, and progression in 7% of the patients [86]. The median treatment duration was 16 months. Hearing improved in 20%, remained stable in 69%, and worsened in 6 percent. The incidence of serious toxicity was 17%, with amenorrhea (70%), proteinuria (43%), and hypertension (33%). Although the dose and schedule of bevacizumab has not been standardized, a proposed regimen is 5–7.5 mg/kg every 2–3 weeks for at least 6 months, followed by maintenance therapy at 2.5–5 mg/kg every 4 weeks [85]. A higher dose regimen (10 mg/kg every 2 weeks for 6 months) offered no clear advantage compared with lower-dose regimens [87], and furthermore higher dose may increase the risk of renal impairment.

Contrarily, problems arise after administration of bevacizumab such as the need for frequent administration, occurrence of hypertension and thrombosis, and apparent drug resistance. Tumor growth rebound following bevacizumab treatment discontinuation was pointed out [88]. Recently, a clinical trial using VEGFR1/2 peptide vaccine was also conducted in patients with progressive NF2-derived schwannomas, showing hearing improvement and tumor volume reduction [89].

The NF2 gene product involves multiple molecular pathways in cell growth. Some studies reported on the efficacy of everolimus, an oral inhibitor of the mTORC1, for progressive vestibular schwannomas in NF2 patients [90,91]. Lapatinib showed objective activity in 4 out of 17 patients with NF2-related progressive vestibular schwannoma in a phase II trial [92], whereas erlotinib was not effective in a retrospective series with 11 NF2 patients [93]. Mirdametinib (PD-0325901) is an orally delivered inhibitor of the dual specificity kinases (MEK1 and MEK2), which demonstrated tumor shrinkage and sustained inhibition of pERK. Furthermore, the recent study demonstrated that hypoxia was significantly associated with shorter progression-free survival in NF2 schwannomas [94]. HIF-1-targeted therapy might be considered for some NF2 schwannomas that are difficult to treat by surgical resection and stereotactic radiosurgery [94].

For patients with severe hearing impairment, strategies using cochlear or brainstem implants may offer some benefit [95,96]. Pilot studies have demonstrated the feasibility of novel psychosocial interventions delivered to deaf individuals with NF2 via teleconferencing with captioning technology [97].

NF2 patients tend to develop meningiomas at an earlier age than those with sporadic meningiomas [98]. The meningiomas seen in NF2 patients are more frequently atypical or anaplastic compared with sporadic tumors [99]. Although radiation therapy has been performed in those patients, long-term follow-up is lacking. Targeted therapies are under investigation [100]. Lapatinib showed some activity in a small number of patients with NF2-associated progressive meningiomas [101]. There is little evidence that bevacizumab has activity in NF2-related meningiomas [102]. These molecular studies led to clinical trials using mTORC1 inhibitor everolimus, a rapamycin analog, for NF2 and sporadic meningiomas. Alternate treatment options for NF2 tumors include inhibitors of the epidermal growth factor receptor, an inhibitor of platelet-derived growth factor, and an inhibitor of histone deacetylase [100].

### 3.4. Ongoing Clinical Trials

Table 4 shows ongoing phase II clinical trials of NF2 patients using various molecular targeted agents besides bevacizumab: icotinib (EGFR inhibitor), axitinib (VEGFR inhibitor), everolimus (mTOR1 inhibitor), crizotinib (c-Met and ALK inhibitor), vistusertib (dual mTORC1/2 inhibitor), brigatinib (ALK and EGFR inhibitor), or selumetinib (MEK inhibitor). Primary outcome is volumetric response or hearing response. Recently, the first phase III randomized clinical trial using bevacizumab was conducted in Japan [103].

### 3.5. Animal Models

In the Nf2 mutant allele, the 3′ part of exon 2 up to the 5′ part of intron 3 has been replaced by a selection marker [104]. Two different mutant Nf2 alleles were generated [105]. One mutant allele, Nf2^KO3^, was generated by an insertional mutation in exon 3. The other mutant allele, Nf2^Δ2^, carried an in-frame deletion of exon 2. Tissue-specific Nf2 inactivation can be accomplished by the Cre/LoxP technology. LoxP recombinatorial sequences are inserted into noncoding regions flanking exon 2 of the Nf2 gene to produce phenotypically normal Nf2 flox mice. Tissue-specific inactivation is mediated by the expression of the bacteriophage Cre recombinase from a tissue-specific promoter or by direct injection of adenoviral Cre [106].

Recently, Chen et al. described the injection of schwannoma cells into the mouse brain cerebellopontine angle region and the application intravital imaging and hearing assessment techniques to study tumor growth and hearing loss. In addition, ataxia, angiogenesis, and tumor–stroma interaction assays could be shown [107].

To date, models of NF2-associated ependymoma remain yet to be generated whereas genetically engineered mouse strain of meningiomas have been. Somatic Nf2 loss after subarachnoid or subdural viral injection of Cre recombinase into newborn Nf2 flox/flox conditional knockout mice result in meningioma [106]. More aggressive tumors develop when NF2 loss is coupled with the loss of other tumor suppressor genes (Ink4a) [108]. It has been challenging to maintain NF-associated tumors as patient-derived xenografts. To date, successful orthotopic transplant models have only been developed for NF2-associated meningioma [109].

## 4. Schwannomatosis

### 4.1. Clinical Characteristics

SWN is the rarest form of NF characterized by multiple schwannomas in the absence of bilateral vestibular schwannomas inherited via autosomal dominance in 15–20% [2,110] (Table 5). Patients with SWN have a median tumor count of 4, and a median whole body tumor volume of 39 mL [111]. The incidence of SWN have ranged from 1 in 40,000 to 1 in 1.7 million people. The median age at diagnosis is approximately 40 years [112], with chronic pain, numbness, tingling, and weakness appearing in early adulthood. Chronic pain is localized or diffused and often does not correlate with the location of schwannomas. Total tumor burden, size, and location do not correlate with pain-related morbidity [113]. While life expectancy of patients with SWN is normal [110,112,113], more data are needed to understand the risk of MPNST and other malignancies in patients with SWN [112,114]. Patients with SWN do not have learning disabilities [113,115]. The incidence of meningioma is 5% [116].

### 4.2. Genetic and Molecular Characteristics

Mutations in SMARCB1 and LZTR1 genes cause SWN. Causative inactivating germline mutations in the tumor suppressor genes SMARCB1 and LZTR1 are present in approximately 85% of families with SWN and up to 40% of sporadic cases. Mutations in the SMARCB1 or LZTR1 gene alone are not sufficient to trigger SWN and require additional somatic mutations. Genetic testing is available for both SMARCB1 and LZTR1 [117].

**Figure 3:**
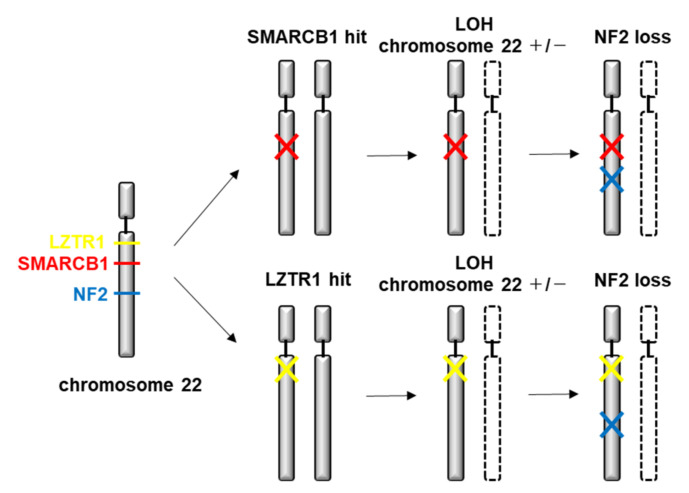
Molecular pathogenesis of SWN.

In SMARCB1 (INI1) mutation-positive schwannomas, there can be additional genetic alterations, including loss of one copy of chromosome 22 and inactivating mutations in the NF2 gene [118,119,120,121,122,123]. This suggests a four-hit, three-step model of tumorigenesis in considerable SMARCB1-associated SWN patients. The mutated germline SMARCB1 gene copy is retained in the tumor (hit 1), whereas chromosome 22, or at least a segment of chromosome 22 containing the wildtype SMARCB1 gene copy and a wildtype copy of the NF2 gene is lost (hits 2 and 3), followed by a mutation in the remaining wildtype NF2 gene copy (hit 4) [119,121,124] (Figure 3). Germline mutations in SMARCB1 can cause an inherited predisposition to atypical teratoid/rhabdoid tumors (ATRT) of the central nervous system [125]. Reports of families with both SWN and rhabdoid tumor phenotypes are rare [114,126,127]. Mosaic loss of immunohistochemical expression of SMARCB1/INI1 is a reliable marker of SWN and can assist in distinguishing SWN from an isolated schwannoma [128]. The presence of abundant myxoid stroma or a hybrid tumor may be also associated with an underlying syndromic diagnosis [124].

Mutations in another tumor suppressor gene, LZTR1, may explain the predisposition to SWN in patients without mutations in SMARCB1 [129,130]. LZTR1 is located on chromosome 22q11.21, centromeric to both SMARCB1 (22q11.23) and NF2 (22q12.2) [129,131]. A larger study on SMARCB1 and NF2 mutation-negative patients revealed LZTR1 mutations in 6 of 16 patients with familial SWN (38%), 11 of 49 sporadic patients (22%), and 2 of 39 patients with unilateral vestibular schwannoma. Somatic LZTR1 mutations have also been found in several other cancers [129,132].

Although biallelic mutations of SMARCB1 or LZTR1 have been detected in the patients with SWN, the classical two-hit model of tumorigenesis is insufficient to account for schwannoma growth, since NF2 is frequently inactivated in these tumors. Tumorigenesis in SWN involves the mutation of at least two different tumor suppressor genes, an occurrence frequently mediated by loss of heterozygosity of large parts of chromosome 22q harboring not only SMARCB1 and LZTR1 but also NF2.

### 4.3. Therapeutic Strategies

To date, there are no established medical therapies that target the SWN, and the use of gabapentin or pregabalin and short-acting opioids and/or nonsteroidal anti-inflammatories has been reportedly successful in reducing pain in patients with SWN. Additional agents, including tricyclic antidepressants such as amitriptyline, serotonin-norepinephrine reuptake inhibitors such as duloxetine, or antiepileptics such as topiramate or carbamazepine may be used as adjuncts or independently. Surgical resection of painful schwannomas may be considered if pain is not successfully controlled. Other surgical indications include spinal cord compression or impingement of other organs.

Both SMARCB1 and LZTR1 interact with histone deacetylase 4 [133,134] which indicates therapeutic implications in SWN. Histone deacetylase inhibitors are currently under development as antitumor drugs [133,134].

### 4.4. Ongoing Clinical Trials

Table 6 shows ongoing clinical trials for SWN patients. Tanezumab is a monoclonal antibody against nerve growth factor as a treatment for pain, which is undergoing Phase II clinical trials for the treatment of various pain entities in SWN patients. Primary outcome is pain relief.

## 5. Future Direction

Elucidation of the molecular pathogenesis of the NF gene has given yield to development of novel targeting strategies. Furthermore, generation of xenograft mouse models of NF associated tumors have enlightened potential molecular targets as well as provided a platform of treatment evaluation prior to human clinical trials. This will likely serve positively for NF patients. Therapeutic trials for the NF will further progress. On the other hand, it is difficult to establish molecular targeted therapy for rare NF-associated tumors (e.g., optic pathway and spinal ependymomas), because evaluating their genomic landscaping is challenging, and hence, they are rarely biopsied or surgically removed. Drugs targeting the pathogenetics will lead to vast advance of disease control.

## Figures and Tables

**Table 1:** Diagnostic criteria of neurofibromatosis type 1.

**A: The Diagnostic Criteria for NF1 Are Met in an Individual Who Does Not Have a Parent Diagnosed with NF1 if Two or More of the Following Are Present:**
At least six café-au-lait macules (>5 mm diameter in prepubertal individuals and >15 mm in postpubertal individuals)
Freckling in axillary or inguinal regions ^#1^
Optic glioma
At least two Lisch nodules identified by slit lamp examination or two or more choroidal abnormalities—defined as bright, patchy nodules imaged by optical coherence tomography/near-infrared reflectance imaging
At least two neurofibromas of any type, or one plexiform neurofibroma
A distinctive osseous lesion such as sphenoid dysplasia, ^#2^ anterolateral bowing of the tibia, or pseudarthrosis of a long bone
A heterozygous pathogenic NF1 variant with a variant allele fraction of 50% in apparently normal tissue such as white blood cells
**B: A child of a parent who meets the diagnostic criteria specified in A merits a diagnosis of NF1 if one or more of the criteria in A are present**

^#1^ If only café-au-lait macules and freckling are present, the diagnosis is most likely NF1 but exceptionally the person might have another diagnosis such as Legius syndrome. At least one of the two pigmentary findings (café-au-lait macules or freckling) should be bilateral. ^#2^ Sphenoid wing dysplasia is not a separate criterion in case of an ipsilateral orbital plexiform neurofibroma.

**Table 2:** Ongoing clinical trials for the patients with NF1.

ID	Initiation Date	Phase	Nation	N	Disease	Treatment	Primary Outcome
NCT04495127	8, 2020	1	Japan	12	NF1	Selumetinib	Toxicity
NCT01968590	8, 2017	2	USA	320	NF1	Cholecalciferol	Bone mineral density
NCT03962543	9, 2019	2	USA	100	NF1 Plexiform Neurofibroma	Mirdametinib (PD-0325901) oral capsule	Complete or partial response rate compared to baseline.
NCT03231306	11, 2017	2	USA	40	NF1 Plexiform Neurofibroma	Binimetinib	Change from Baseline Target Tumor Volume at 12 months
NCT02839720	4, 2017	2	USA	24	Cutaneous Neurofibroma NF1 Optic Nerve Glioma	Selumetinib	Change in the size
NCT02407405	1, 2016	2	USA	60	NF1 Plexiform Neurofibromas	Selumetinib	Determine objective response rate
NCT04461886	7, 2020	3	Japan	100	NF	NPC-12G gel	Discontinuation rate associated with adverse events
NCT03871257	10, 2019	3	USA	290	Low Grade Glioma NF1 Visual Pathway Glioma	Carboplatin Selumetinib Sulfate Vincristine Sulfate	Event-free survival
NCT02101736	6, 2014	2	USA	48	NF1 Neurofibromatosis Plexiform Neurofibromas	Cabozantinib	The change in tumor size based on radiographic assessment
NCT03326388	9, 2019	1/2	USA	30	NF1 Plexiform Neurofibroma Optic Nerve Glioma	Selumetinib	To evaluate the Maximum Tolerated Dose Objective response rate
NCT03741101	6, 2019	2	Sweden	15	NF1 Plexiform Neurofibromas	Trametinib	Remission of tumor volume ≥20%
NCT02728388	8, 2016	2	USA	30	NF1	aminolevulinic acid	Time to disease progression
NCT04435665	8, 2020	2	USA	48	NF1 Cutaneous Neurofibroma	NFX-179 Gel	Phospho-erk (p-ERK) levels of Target cNF Tumors Toxicity
NCT02390752	4, 2015	1/2	USA	81	Neurofibroma, Plexiform	PLX3397	Toxicity Objective response rate
NCT03688568	9, 2018	2	USA	20	Neurofibroma, Plexiform	Imatinib Mesylate	Quantitative Functional Airway Response
NCT03433183	10, 2019	2	USA	21	Malignant Peripheral Nerve Sheath Tumors NF1	Selumetinib Sirolimus	Clinical benefit rate of selumetinib in combination with sirolimus
NCT04085159	9, 2019	1/2	China	100	Neurofibromatosis Schwannomatosis	Antigen-specific T cells CART/CTL and DCvac	Percentage of adverse effects

NF1, neurofibromatosis type 1.

**Table 3:** Diagnostic criteria of neurofibromatosis type 2.

Bilateral vestibular schwannomas or
First-degree relative with neurofibromatosis type 2 plus
1. Unilateral vestibular schwannomas or
2. Any two of the following: Meningioma, glioma, schwannoma, or juvenile PLO

PLO, posterior lenticular opacities.

**Table 4:** Ongoing clinical trials for the patients with NF2.

ID	Initiation Date	Phase	Nation	N	Disease	Treatment	Primary Outcome
NCT02934256	7, 2016	2	China	20	NF2	Icotinib	Change from Baseline in volume of tumor
NCT02129647	4, 2014	2	USA	12	NF2 Progressive VS	Axitinib	volumetric response rates
NCT01345136	7, 2015	2	USA	4	NF2	RAD001, everolimus	Vestibular schwannoma volume
NCT01767792	5, 2013	2	USA	22	NF2 Progressive VS	Bevacizumab	Hearing
NCT04283669	2, 2020	2	USA	19	NF2 Progressive VS	Crizotinib	Volumetric response rate
NCT02831257	8, 2016	2	USA	18	NF2 Meningioma	AZD2014	Volumetric response rate
NCT04374305	6, 2020	2	USA	80	NF2 Vestibular Schwannoma Non-vestibular Schwannoma Meningioma Ependymoma	Brigatinib	Volumetric response rate
NCT03095248	5, 2017	2	USA	34	NF2 Vestibular Schwannoma Meningioma Ependymoma Glioma	Selumetinib	Hearing response Volumetric response rate
NCT03079999	6, 2018	2	USA	300	NF2 Vestibular schwannoma	Aspirin	Progression-free survival

NF2, neurofibromatosis type 2.

**Table 5:** Diagnostic criteria of schwannomatosis.

**Definite Schwannomatosis**
A. Age >30 years and two or more schwannomas (not intradermal), at least one with histologic confirmation with no evidence of vestibular tumor on brain MRI scan and no known NF mutation
B. Vestibular schwannoma (pathologically confirmed) plus first-degree relative who meets the criteria of schwannomatosis
**Possible schwannomatosis**
A. Age <30 years plus two or more schwannomas (not intradermal), at least one with histologic confirmation with no evidence of vestibular tumor on brain MRI scan and no known NF mutation
B. Age >45 years plus two or more schwannomas (not dermal), at least one with histologic confirmation and no symptoms of 8th nerve dysfunction and NF type 2
C. Evidence of a non-vestibular schwannoma and first-degree relative meeting criteria for definite schwannomatosis

MRI, magnetic resonance imaging; NF, neurofibromatosis.

**Table 6:** Ongoing clinical trials for the patients with schwannomatosis.

ID	Initiation Date	Phase	Nation	N	Disease	Treatment	Primary Outcome
NCT04163419	4, 2020	2	USA	46	Schwannomatosis	Tanezumab	Change in pain level
NCT04085159	9, 2019	1/2	China	100	Neurofibromatosis Schwannomatosis	Antigen-specific T cells CART/CTL and DCvac	Percentage of adverse effects

## Data Availability

Not applicable.
